# Laboratory investigation of high pressure survival in *Shewanella oneidensis* MR-1 into the gigapascal pressure range

**DOI:** 10.3389/fmicb.2014.00612

**Published:** 2014-11-17

**Authors:** Rachael Hazael, Fabrizia Foglia, Liya Kardzhaliyska, Isabelle Daniel, Filip Meersman, Paul McMillan

**Affiliations:** ^1^Christopher Ingold Laboratories, Department of Chemistry, University College LondonLondon, UK; ^2^Laboratoire de Géologie de Lyon, UMR 5276 CNRS, ENS de Lyon and Université Claude Bernard Lyon 1 – Université de LyonLyon, France; ^3^Biomolecular and Analytical Mass Spectrometry group, Department of Chemistry, University of AntwerpAntwerpen, Belgium

**Keywords:** high pressure biology, *Shewanella oneidensis* MR-1, bacterial survival, piston cylinder experiments, pressure adaptation studies, variable temperature bacterial growth

## Abstract

The survival of *Shewanella oneidensis* MR-1 at up to 1500 MPa was investigated by laboratory studies involving exposure to high pressure followed by evaluation of survivors as the number (*N*) of colony forming units (CFU) that could be cultured following recovery to ambient conditions. Exposing the wild type (WT) bacteria to 250 MPa resulted in only a minor (0.7 log *N* units) drop in survival compared with the initial concentration of 10^8^ cells/ml. Raising the pressure to above 500 MPa caused a large reduction in the number of viable cells observed following recovery to ambient pressure. Additional pressure increase caused a further decrease in survivability, with approximately 10^2^ CFU/ml recorded following exposure to 1000 MPa (1 GPa) and 1.5 GPa. Pressurizing samples from colonies resuscitated from survivors that had been previously exposed to high pressure resulted in substantially greater survivor counts. Experiments were carried out to examine potential interactions between pressure and temperature variables in determining bacterial survival. One generation of survivors previously exposed to 1 GPa was compared with WT samples to investigate survival between 37 and 8°C. The results did not reveal any coupling between acquired high pressure resistance and temperature effects on growth.

## INTRODUCTION

The identification of organisms surviving in deep subsurface habitats has raised important questions concerning the absolute limits of life forms exposed to extreme stresses, including high pressure (Bartlett, 2002; Oger and Jebbar, 2010; Picard and Daniel, 2014). Since the early work of ZoBell and Johnson (1949) a wide range of laboratory and field studies have shown that the survival, growth, and metabolism of surface dwelling species and piezophile organisms are significantly affected by exposure to high pressure conditions (Bartlett, 2002; Colwell and D’Hondt, 2013). Explorations of deep oceanic and continental subsurface habitats have returned organisms whose habitat is at pressures up to 110 MPa (Edwards et al., 2012; Picard and Daniel, 2014). In most environments the limit for microbial life at depths might not be determined by an upper pressure boundary but by the rise in temperature above approximately 120°C due to the geotherm (Oger and Jebbar, 2010; Picard and Daniel, 2014). Living organisms might be encountered at higher pressures in deep, cold environments on Earth such as subduction zones as well as other icy planetary bodies like Enceladus, Titan, or Ganymede, so that it is important to investigate the upper limits to bacterial survival as well as the mechanisms that microorganisms employ to counteract extreme pressurization effects. Such work is also important for sterilization processes employing “Pascalization” (high P) vs. “Pasteurization” (high T) techniques that are increasingly used to preserve color, flavor, texture, and nutritional value among food-related products (Hite et al., 1914; Van Opstal et al., 2005; Aertsen et al., 2009; Demazeau and Rivalain, 2011; Meersman et al., 2013). Experimental studies have begun to investigate the ultimate pressure limits to bacterial survival, both as a result of initial compression of naturally occurring wild type (WT) microorganisms, and following resuscitation of survivors from initial pressurization experiments, that can reveal either adaptation of parts of the population to extreme compression or the elimination of individuals that were less able to resist extreme pressurization.

In a pioneering study Sharma et al. (2002) suggested that bacteria including *E. coli* and *S. oneidensis* MR-1 continued to survive and metabolize at pressures up to 1.4 GPa, that greatly exceeded the range previously considered for possible pressure limits to life. However, those results and their interpretation were criticized within the microbiological community who noted that the data might simply represent the continuation of enzymatic activity persisting after cell death (Yayanos, 2002; Picard and Daniel, 2014). Our group recently carried out an investigation of *E. coli* survival into the GPa range, using colony formation and counting techniques for samples recovered following high pressure exposure, along with sequential pressure treatment of samples taken from survivor colonies following resuscitation (Vanlint et al., 2011). The results showed no detectable survivors or evidence for colony formation from the WT population following applied pressure stress of 700 MPa or above. However, we did observe significant (1–2%) survival following pressurization extending up to 2 GPa, for colonies derived from survivor populations that were resuscitated following multiple exposures to increasing pressure conditions (Hauben et al., 1997; Vanlint et al., 2011). That result could indicate that some fraction of the initial *E. coli* population contained a biomolecular mechanism to resist such extreme high pressure conditions, and had the ability to be resuscitated and grow upon returning to ambient pressure. This characteristic might be inherited among successive generations following systematic exposure to successively higher pressures. An alternative interpretation is that development of pressure-resistant characteristics could occur within parts of the population as a function of continued pressurization to higher pressures. Our initial *E. coli* study also examined the possibility of cross-correlation between high P- and high T-resistant traits emerging among the populations (Vanlint et al., 2011). However, those results showed no correlation between these traits.

In the present work we have extended our studies to *S. oneidensis* MR-1, to conduct an investigation of the combined effects of pressure survival, resuscitation and growth temperature effects on the colony-forming behavior of WT vs. “pressurized” survivor strains. This model organism was selected as a member of the *Shewanella* genus that contains both facultative and obligate piezophilic species, e.g., *S. violacea, S. profunda*, and *S. benthica* (Venkateswaran et al., 1999). We followed a similar experimental protocol to our previous *E. coli* study using piston cylinder techniques to expose the microbial samples to pressures extending into the GPa range followed by plate counting of survivors (Vanlint et al., 2011), and assessing their ability to resuscitate and establish colonies.

It is important to investigate possible cross-correlation effects between high pressure and variable temperature survival characteristics. *S. oneidensis* grows optimally at 30°C at ambient pressure (Venkateswaran et al., 1999). Within the *Shewanella* genus several strains grow and thrive in high pressure environments and at cold temperatures. For example *S. benthica* grows best at just over 50 MPa and 10°C (Kato and Nogi, 2001; Lauro et al., 2013) whereas *S. frigidimarina* is best adapted for growth at ambient pressure and 15°C (Bozal et al., 2002). Kato and Nogi (2001) concluded that two sub-genus branches can be considered: *Shewanella* group 1: high-pressure cold-adapted species, and *Shewanella* group 2: characterized as mesophilic pressure-sensitive species. *S. oneidensis* would be classed within group 2 as would *S. frigidimarina*, whereas *S. benthica* would be classified within group 1.

In a first set of studies we carried out all pressurization experiments using the WT strain to establish the intrinsic resistance of a population found under ambient conditions and exposed to extreme high P environments. We then conducted further experiments using survivor colonies established following previous pressurization and resuscitation stages to observe their survival to even higher pressures. Finally, we examined the survival and colony-forming characteristics of WT and P-adapted populations at temperatures extending between 8 and 37°C.

## MATERIALS AND METHODS

*Shewanella oneidensis* MR-1 (CIP 106686) was purchased from the Collection Institut Pasteur (Paris, France) (Venkateswaran et al., 1999). These were subsequently rehydrated in 200 μl of Luria-Bertani Miller (LB) medium. From this 50 μl was used for a liquid culture in 10 ml of LB broth to be grown at 30°C and 180 rpm, and two separate plate spreads of 50 μl each allowed stock solutions to be made.

For each experiment a 10 ml starter culture was inoculated either from a plate or from stock. The bacteria were harvested in stationary phase at a concentration of 4 ± 1 × 10^8^ cells/ml. For each experiment 1 ml aliquot of the starter culture was washed three times with phosphate buffered saline (PBS) solution adjusted to pH 7.2. This allows only live bacteria to be present in the sample, as any components of broken cells are washed out and no cell clumps are present. The high pressure capsule was loaded with 700 μl of the bacterial suspension. An aliquot of this sample was plated to serve as a control for the pressurized bacterial sample. All microbiological preparations and sample handling were carried out under aseptic conditions.

**FIGURE 1:**
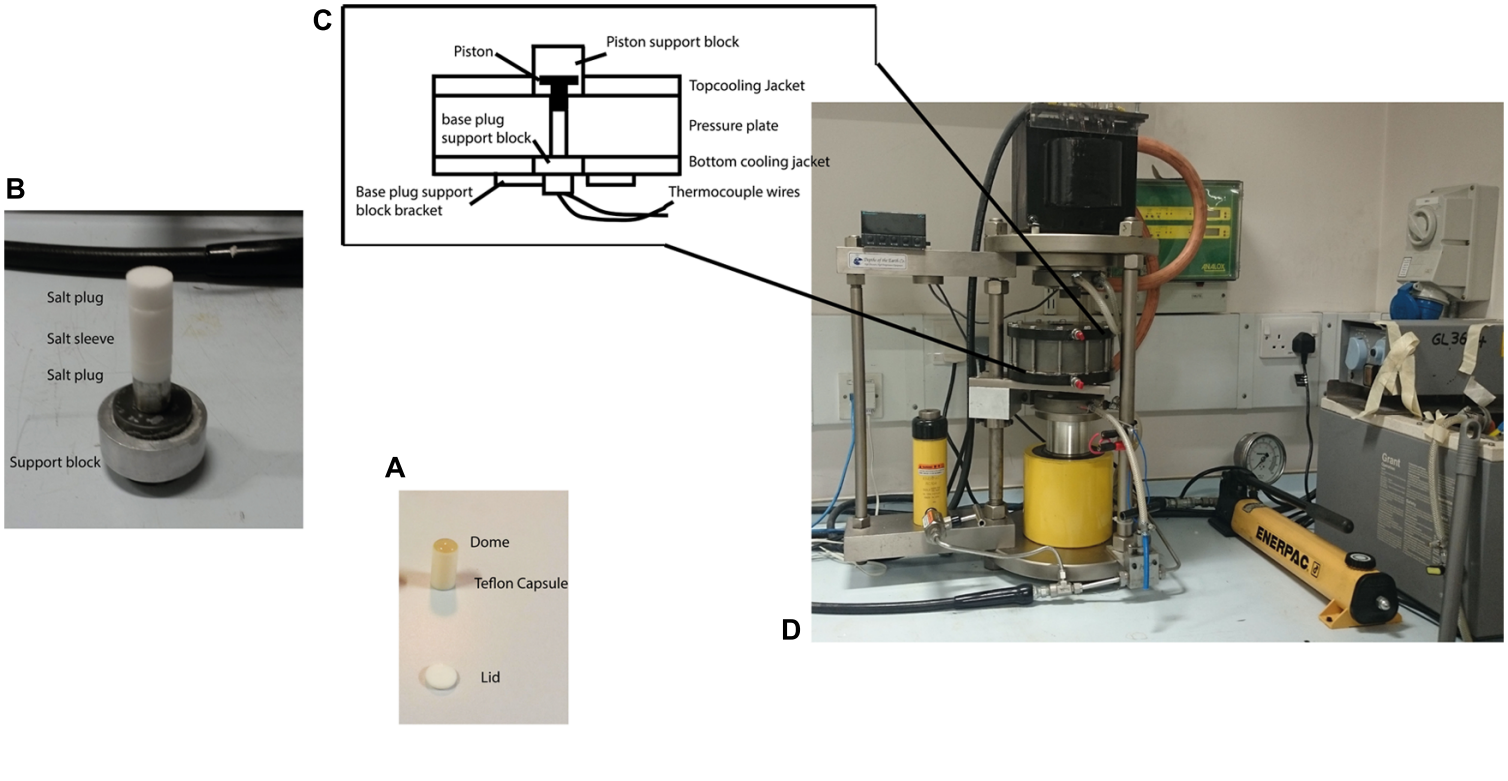
**The piston cylinder device (http://www.depthsoftheearth.com) and protocol used for carrying out the high pressure experiments. (A)** The Teflon^®^ capsule is filled with cell suspension and is capped by a Teflon^®^ lid for the experiment. The dome of liquid indicates the capsule is completely filled and contains no air. **(B)** The capsule is placed inside a pre-formed cylinder (sleeve) made from pressed powdered NaCl that acts as a pressure-transmitting medium with NaCl plugs capping both ends. **(C)** The sample assembly is then placed inside the central bore of the pressure plate. The tungsten carbide piston is inserted from the top. Thermocouple wires inserted from the bottom and passing through the lower pressure support block to make contact with the sample capsule were used to measure the temperature. **(D)** Pressure is applied by hand pumping the oil-driven hydraulic ram to raise the pressure plate containing the sample assembly and force it against the piston that is driven down to reduce the sample volume. The pressure is transmitted to the sample through slight deformation of the walls of the Teflon^®^ container that maintains its shape and remains intact during the experiment and subsequent recovery.

Pressurization experiments on the microbial suspensions were carried out using a piston cylinder device typically used for experiments in Earth and solid state sciences (**Figure 1**). This enables high pressure studies to be carried out to >3 GPa with sample volumes extending up to approximately 700 μl. This provided sufficient material to ascertain bacterial survival *via* plate counting techniques on recovered samples, as well as providing samples for further imaging, spectroscopic, and culturing experiments. The bacterial suspensions were loaded into Teflon^®^ capsules (Hill et al., 2011; Vanlint et al., 2011) and placed inside a sleeve pre-formed from powdered NaCl that acted as a pressure-transmitting medium. The sample assembly was mounted inside the bore of the pressure plate and the piston introduced from the top. Pressure was then increased by hand pumping a hydraulic ram that allowed the target pressures to be obtained within a few seconds. This is negligible in comparison with the timescale of the pressurization experiments that were typically held at target pressure for 15 min. During the mechanical compression the salt sleeve undergoes plastic flow and provides an approximately hydrostatic pressure transmitting environment surrounding the sample chamber. For variable temperature studies a water bath was constructed using various ice/water mixtures to control the inlet temperature of the cooling coils that surrounded the piston cylinder pressure plate. The sample temperature was monitored by a K-type thermocouple inserted into the base of the sample holder. After calibration the apparatus achieved temperature stability within ±1°C over a 30–60 min period spanning the duration of each experimental run. Once the desired P, T conditions were reached the sample was held for 15 min before recovery to ambient conditions, removal from the capsule and subsequently plating and/or otherwise characterizing the bacterial culture obtained. Holding times in the 10–15 min range are typically used in high-pressure food microbiology studies as a convenient compromise between various experimental and biological parameters. In previous work on *E. coli* we found that different pressure-resistant mutants exhibited differences in inactivation kinetics such that the log (*N*) survival counts became indistinguishable for pressurization runs of 45 min or more (Vanlint et al., 2011). Selecting 15 min as a run duration allowed a clear distinction between pressure survival characteristics while being substantially long compared with pressure run-up and equilibration times (on the order of <1 min), and remaining within feasible bounds for conducting multiple pressurization-recovery experiments. Kish et al. (2012) found that the *E. coli* MG1655 strain showed a substantial decrease in survival rate (*N*/*N*_o_) in pressure survival experiments at 400 MPa carried out for 60 min, whereas *Halobacterium salinarum* NRC-1 showed no change in survival at the same pressure, over the same range of experimental run durations. Establishing the relationships between inactivation kinetics and bacterial survival rates as a function of pressure and temperature variables, for different WT vs. pressure-adapted microbial strains, is an important topic to be examined in future work.

The WT bacteria were exposed to pressures extending up to 1.5 GPa (1500 MPa) at 21°C. Upon decompression to ambient conditions the suspensions were recovered from the capsule and plated onto LB Miller agar following sixfold dilution. Once colonies had developed the number of cells per ml (*N*) were determined using plate counting. Most experiments were conducted at least in triplicate (**Table 1**).

**Table 1:** Results of *Shewanella oneidensis* survival experiments as a function of applied pressure stress at constant temperature (21°C).

Previous pressure(s) experienced/MPa	Pressure/MPa	Survivor counts/CFU/ml	Average N/CFU/ml (SD)
None (WT)	250	9, 10, 9 (×10^7^)	9.3 × 10^7^ (60)
None (WT)	500	150, 140, 155 (×10^2^)	1.5 × 10^4^ (8)
None (WT)	750	132, 130, 155 (×10^2^)	1.4 × 10^4^ (14)
None (WT)	1000	140, 927^a^	5.2 × 10^2^
None (WT)	1500	132, 132, 130^a^	1.31 × 10^2^ (1)
250	500	25, 24, 24 (×10^5^)	2.4 × 10^6^ (6)
250 and 500	750	2, 2, 2 (×10^7^)	2.1 × 10^7^ (11)
250 and 500 and 750	1000	22 (×10^5^)	2.2 × 10^6^
250 and 500 and 750	1500	5, 6, 3 (×10^5^)	4.6 × 10^5^ (153)

*Starting average concentration for WT experiments was 10^*8*±*0.5*^ cells/ml. The decadal exponent in brackets in the survivor counts column refers to the number of successive dilutions carried out. The value in parentheses following the mean average survivor count indicates 1 SD for the nine data points obtained for each sample. For two samples exposed to 1000 MPa insufficient count data were obtained to carry out a statistical test. Each experiment was held at pressure for a duration of 15 min. ^a^These CFU/ml values were determined *via* plate spreads followed by microscopic examination with no dilution factor*.

To study effects of previous pressure exposure on survival the bacteria were first exposed to an initial pressure value followed by decompression to ambient where the survivors were re-cultured to obtain colonies. One colony picked randomly from the plated survivors was then used to inoculate a starter culture that was taken to a next highest pressure, e.g., 500 MPa. That process was repeated sequentially with subsequent survivor cultures exposed to progressively higher pressures, 750 MPa and then 1000 MPa. In our highest pressure experiment, samples obtained from the 750 MPa survivor culture were taken directly to 1500 MPa.

**FIGURE 2:**
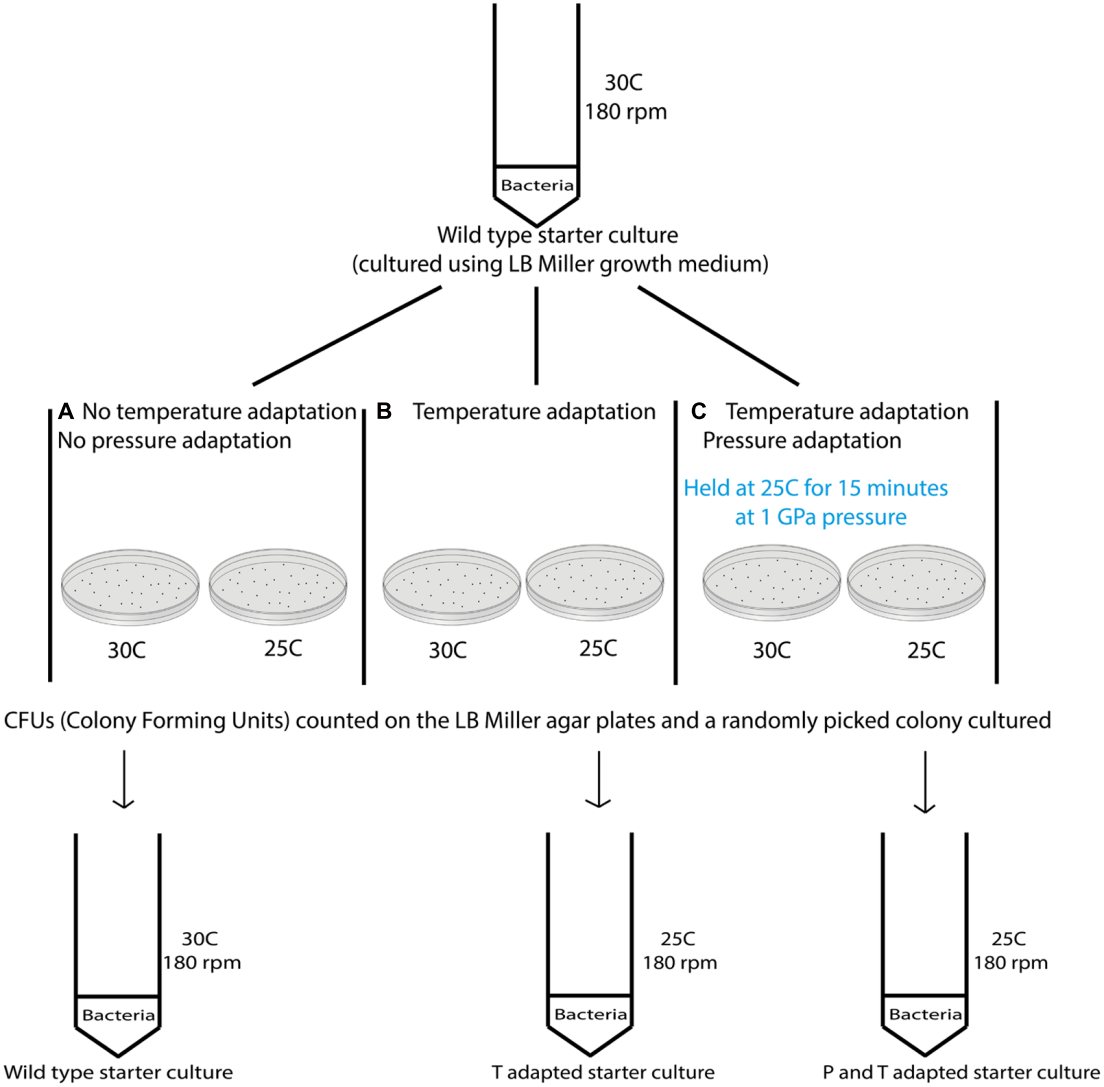
**Schematic showing the different steps examined in the variable temperature growth and exposure experiments.** Three different treatment protocols are indicated in the figure. Route **(A)** provides a control experiment for samples exposed to routes **(B,C)**. In route **(A)** cultures that had previous experienced only ambient pressure and temperature conditions were grown at 30°C while being rotated at 180 rpm. Once a pellet was obtained the sample was plated and aliquots were incubated at the different temperatures investigated (i.e., 37, 30, 25, 19, and 8°C). Colonies formed were counted and the results are shown in **Table 2**. Route **(B)** cultures were obtained from survivors of the wild type (WT) strain that had been exposed to 1000 MPa ( **Table 1**). These were cultured at 30°C and 180 rpm from stocks created from the survivor population. Each sample was then incubated at different temperatures (37, 30, 25, 19, and 8°C). Route **(C)** cultures were likewise isolated from survivors of the initial 1000 MPa exposure experiment, cultured at 30°C and 180 rpm from stocks. In the next step these samples were exposed to different temperatures (30, 25, 19, or 8°C) while being held once more at high pressure (1000 MPa).

Variable temperature experiments were conducted using the protocol depicted in **Figure 2**. In a first series of studies (route A) a 10 ml starter culture of wild type *S. oneidensis* initially grown to stationary phase at 30°C and 180 rpm was plated onto LB Miller agar and placed in ovens held at 30°C as well as the next lower temperature in the adaptation study, e.g., 25°C to observe colony formation at these temperatures (**Figure 2**). To compare the growth at different temperatures with survivors from extreme pressurization a 10 ml starter culture was then obtained from isolates from an experiment in which the WT strain was taken directly to 1000 MPa (**Table 1**). The isolates were plated and incubated in separate ovens at 30 and 25°C (route B). In a third series of experiments (route C) the isolates from the 1000 MPa survivors were grown in a 10 ml starter culture, returned to 1000 MPa pressure and held for 15 min. While under pressure the samples were simultaneously cooled to a lower temperature value, beginning with 25°C. Following recovery, the samples were plated onto LB Miller agar and incubated at either 30 or 25°C as before. In a final set of runs, we attempted a directed temperature study similar in protocol for the pressurization experiments. From the LB Miller agar plate incubated from the 1 GPa pressure survivors at 25°C, a colony was isolated, cultured, prepared as above and then re-exposed where it was held for 15 min at a lower temperature (e.g., 19°C this was then repeated and the temperature decreased further to 8°C).

## RESULTS

### SURVIVABILITY FOLLOWING PRESSURIZATION AT ROOM TEMPERATURE

**FIGURE 3:**
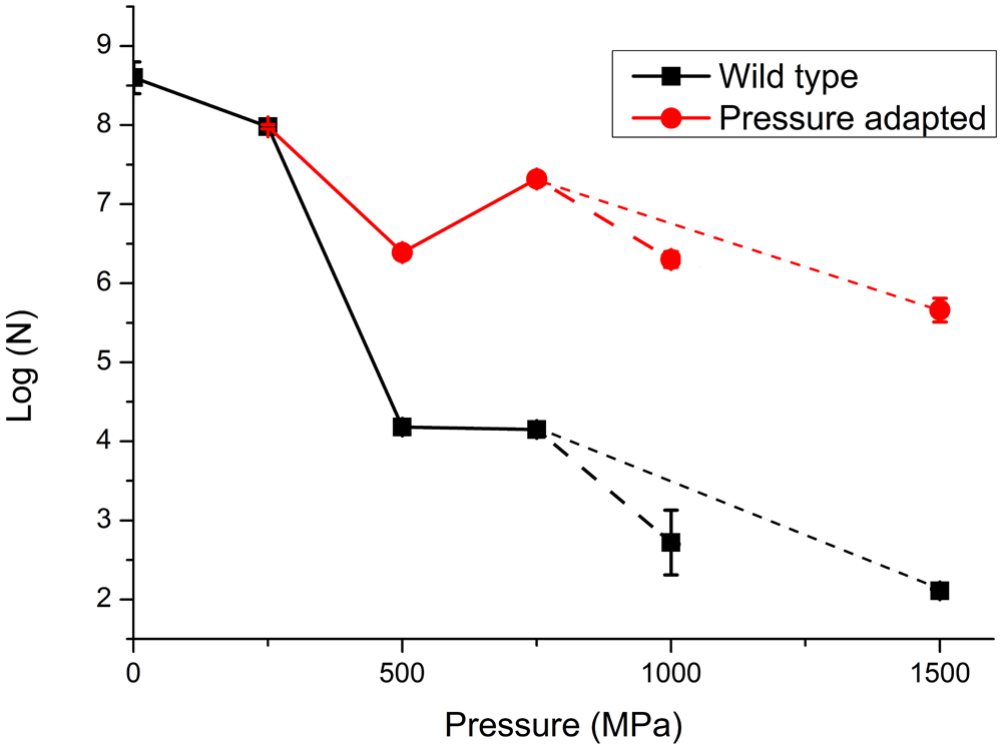
**Survivability of *S. oneidensis* MR-1 following exposure to high pressure extending up to 1000 MPa (1 GPa).** Data are reported on a logarithmic scale with *N* as the number of CFUs per ml determined following recovery to ambient pressure. Data points represent averaged results from multiple experiments: individual run results are listed in **Table 1**. The point at 0.1 MPa (ambient pressure) represents the starting solution with initial concentration 10^8±0.5^ cells/ml. The lines are sketched to show a link between data points. In a first series of experiments the WT strain was taken directly to each pressure of 250, 500, 750, and 1000 MPa in single steps. In further experiments the survivors following pressure exposure were cultured to form a “pressure adapted” population, that was then exposed to a next higher pressure step. The sequential process of pressure exposure, survivor culture, then subsequent exposure to higher pressure was repeated several times for different samples. Dashed lines extending to 1 GPa (larger dashed lines) and 1.5 GPa (smaller dashed lines) points represent different pathways for pressure exposure, survivor culture that were taken after 750 MPa, (pathway 1: 250, 500, 750, and 1000 MPa and pathway 2: 250, 500, 750, and 1500 MPa). Details of individual experiments are given in **Table 1**. Size of the data marker and error bars indicate the range of CFUs counted for each data point.

In a first series of experiments, wild type *S. oneidensis* MR-1 bacteria were taken directly to pressures of 250, 500, 750, 1000, and 1500 MPa and recovered to ambient conditions. The number of survivors following each pressurization experiment was determined as the number of colony forming units (CFU) per ml (*N*; **Table 1**; **Figure 3**). The numbers of surviving CFU/ml dropped rapidly above 250 MPa with a decrease of 4–5 log (*N*) units by 500 MPa. By 1000 MPa only 130–140 cells/ml could be detected in the recovered sample; however, those highly P-resistant individuals could be successfully cultured and they were used for the pressure-temperature cross-correlation studies described in the next section (**Table 1**). Although no statistical analysis is possible from the limited data set the variation in *N* values between individual runs gives an indication of the reproducibility, and the spread in values is used to estimate the error bars shown in **Figure 3**.

At the highest pressure achieved in this study (1500 MPa) our results demonstrated that a few cells were able to survive this extreme pressurization treatment and form colonies following recovery to ambient. These survivors (WT) showed a 6 log unit reduction from the original starting concentration, but only a 3.5 log unit reduction in comparison with resuscitated “P-adapted” survivor colonies that had also been taken to 1500 MPa (**Table 1**; **Figure 3**).

In a next series of pressure survival experiments we added resuscitation steps to investigate the possibility of “directed evolution” following initial exposure to high pressure conditions (Vanlint et al., 2011). The results shown in **Figure 3** compare data for the wild type strain taken directly to each pressure with experimental runs in which survivors from an initial pressure exposure (e.g., 250 MPa) were plated and re-cultured. From **Figure 3** it is evident that the bacteria cultured following the pressurization-resuscitation protocol had a significantly higher degree of pressure survival than the WT strain, with the pressurized-resuscitated strain reaching a survival count of four orders of magnitude higher at 1000–1500 MPa. Both data sets appear to show the largest reduction in survival between 250 and 500 MPa. Above that pressure the WT strain continued to be reduced in its number of survivors, but the pressure-adapted strain appeared to increase by approximately 1 log (*N*) unit for the 750 and 1000 MPa pressure points. After 1000 MPa the survival counts showed a reduction to below the 500 MPa survival at 1500 MPa (**Figure 3**).

### COMBINED EFFECTS OF EXPOSURE TO VARIABLE PRESSURES AND TEMPERATURES

**FIGURE 4:**
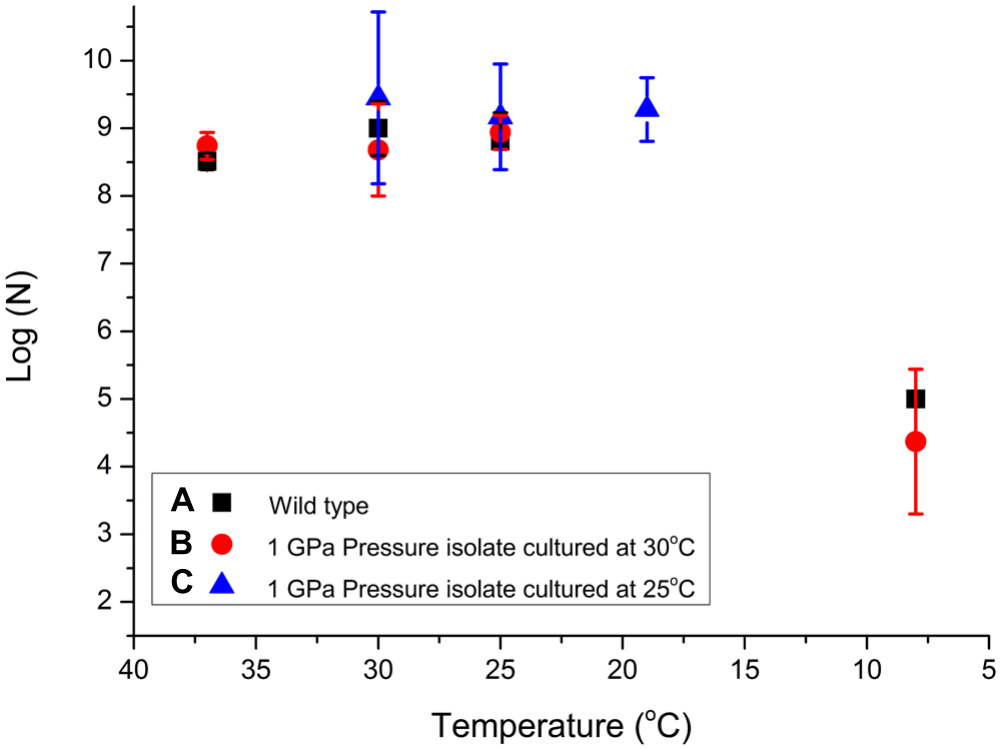
**Examination of *S. oneidensis* survival following exposure to different sets of temperature and pressure conditions to investigate possible cross-correlation effects. Table 2** shows the raw data (*N*) for the T experiments. Data points showing log (*N*) are compared for **(A)** WT bacteria previously cultivated at 30°CC then exposed for 15 min at temperatures between 37 and 8°CC (black squares); **(B)** samples from a colony resuscitated following exposure to 1 GPa pressure, subsequently cultured at 30°C, and then exposed to temperatures between 37 and 8°C (red circles); **(C)** resuscitated samples previously exposed to 1 GPa, cultured at 25°C, then re-exposed to temperatures between 37 and 8°C (blue triangles). Individual run data results are reported in **Table 2**. Size of the data marker and error bars indicate the range of CFUs counted for each data point.

The results of our experiments carried out to investigate potential pressure-temperature cross-correlation effects are detailed in **Table 2** (Supplementary Information). As for the pressurization experiments an initial culture of the starter WT pellet with concentration 10^8±0.5^ cells/ml was grown at various temperatures (**Figure 2**). We observed relatively little variation between 37 and 19°C but the number of viable CFU/ml observed following exposure to 8°C dropped by approximately 4 log units (**Figure 4**). We found no obvious pressure-temperature cross-correlation effect within the same temperature range for the strain that had been previously exposed to 1000 MPa and cultured at 30°C, although that strain appeared to show lower survival at 8°C than the WT strain. In further experiments (see **Figure 2** for a detailed description of the protocols followed) we observed that the high pressure (1000 MPa) survivors cultured at a lower temperature (25°C) and plated at 30–19°C did show a small increase in viability (**Figure 4**). Showing that the change in culturing temperature from 30 to 25°C had a small affect on the viability of the cells. Finally, we attempted several experiments in which the cultivated 1 GPa survivors were simultaneously exposed to a further 1 GPa treatment at various temperatures, but no obvious trend in log (*N*) values could be distinguished (data listed in **Table 2**, but not included in **Figure 4** for clarity).

**Table 2:** Results of *S. oneidensis* growth and exposure experiments at variable temperatures between 37 and 8^**o**^C.

Sample	Inoculation Temperature (°C)	Experiment Temperature (°C)	Pressure (MPa)	Incubation T post-experiment	Survivors (CFU/ml)	Average *N* (CFU/ml)
Wild type (WT)	30	Ambient	Ambient	37	1, 3, 6, 3 (×10^8^)	3.25 ×10^8^
WT	30	Ambient	Ambient	30	15, 11, 5, 9 (×10^8^)	10 × 10^8^
WT	30	Ambient	Ambient	25	8, 8, 7, 3 (×10^8^)	6.5 × 10^8^
WT	30	Ambient	Ambient	8	1, 1, 1, 1 (×10^5^)	1 × 10^5^
1 GPa strain	30	Ambient	Ambient	37	3, 10, 3, 6 (×10^8^)	5.5 × 10^8^
1 GPa strain	30	Ambient	Ambient	30	6, 4, 5,4 (×10^8^)	4.8 × 10^8^
1 GPa strain	30	Ambient	Ambient	25	8, 11, 13, 3 (×10^8^)	8.8 × 10^8^
1 GPa strain	30	Ambient	Ambient	8	21, 25, 24, 23 (×10^3^)	23.2 × 10^3^
1 GPa strain	25	Ambient	Ambient	30	29, 26, 29 (×10^8^)	28 × 10^8^
1 GPa strain^C^	25	Ambient	Ambient	25	12, 17, 16, 14 (×10^8^)	14.8 × 10^8^
1 GPa strain	25	Ambient	Ambient	19	26, 10, 17, 23 (×10^8^)	19 × 10^8^
WT	25	Ambient	Ambient	30	41, 33, 44, 30 (×10^8^)	37 × 10^8^
WT	25	Ambient	Ambient	25	10, 16, 20, 23 (×10^8^)	17.2 × 10^8^
WT	25	Ambient	Ambient	19	31, 25, 25, 30 (×10^8^)	27.8 × 10^8^
WT	25	Ambient	Ambient	8	1,1,1,1 (×10^4^)	1 × 10^4^
WT	19	Ambient	Ambient	19	26, 22, 18, 19 (×10^8^)	21.2 × 10^8^
1 GPa strain	19	Ambient	Ambient	19	19, 12, 10, 17 (×10^8^)	14.5 × 10^8^
1 GPa strain	30	37	1000	37	1,1,1,1 (×10^3^)	1 × 10^3^
1 GPa strain	30	37	1000	30	1,1,1,1 (×10^3^)	1 × 10^3^
1 GPa strain	30	37	1000	25	1,1,1,1 (×10^4^)	1 × 10^3^
1 GPa strain	30	37	1000	8	1,1,1,1 (×10^3^)	1 × 10^3^
1 GPa strain	30	30	1000	37	1,1,1,1 (×10^3^)	1 × 10^3^
1 GPa strain	30	30	1000	30	1,1,1,1 (×10^3^)	1 × 10^3^
1 GPa strain	30	30	1000	25	1,1,1,1 (×10^3^)	1 × 10^3^
1 GPa strain	30	30	1000	8	1,1,1,1 (×10^3^)	1 × 10^3^
WT	30	Ambient	1000	37	1,1,1,1 (×10^3^)	1 × 10^3^
WT	30	Ambient	1000	30	0	0
WT	30	Ambient	1000	25	1,1,1,1 (×10^3^)	1 × 10^3^
WT	30	Ambient	1000	8	0	0
1 GPa strain	25	Ambient	Ambient	25	5, 5, 6, 5 (×10^8^)	5.2 × 10^8^
1 GPa strain	25	Ambient	Ambient	19	9, 14, 15, 10 (×10^6^)	12 × 10^6^
1 GPa strain	25	Ambient	Ambient	19	26, 10, 17, 23 (×10^8^)	19 × 10^8^
1 GPa strain	25	Ambient	Ambient	8	15, 18, 18, 10 (×10^8^)	15.2 × 10^8^
1 GPa strain	19	Ambient	Ambient	19	18, 12, 10, 17 (×10^8^)	14.2 × 10^8^
1 GPa strain	19	Ambient	Ambient	8	4, 2, 9, 3 (×10^6^)	4.5 × 10^6^
1 GPa strain	25	Ambient	Ambient	8	9, 6, 6, 8 (×10^2^)	7.25 × 10^2^
WT	25	Ambient	Ambient	19	23, 16, 16, 21 (×10^8^)	19 × 10^8^
WT	25	Ambient	Ambient	19	31, 25, 25, 30 (×10^8^)	27.8 × 10^8^
WT	25	Ambient	Ambient	25	15, 14, 11, 16 (×10^8^)	14 × 10^8^
WT	19	Ambient	Ambient	19	26, 22, 18, 18 (×10^8^)	21 × 10^8^
WT	25	Ambient	Ambient	8	1,1,1,1 (×10^4^)	1 × 10^4^
WT	19	Ambient	Ambient	8	1,1,1,1 (×10^4^)	1 × 10^4^

*Experiments were carried for (a) WT strain and (b) survivor isolates following an initial pressure stress at 1000 MPa (1 GPa). Ambient T refers to laboratory temperature of 21°CC. Averages were taken over four independent 10 μl spots in each experiment*.

## DISCUSSION

Our experiments show two main results. First, WT samples of *S. oneidensis* MR-1 exhibit a rapid decrease in survival above 250 MPa, with a decrease of 4.5 log units in CFU/ml that can be cultivated following exposure to 500 MPa. The survivor counts continue to decrease further upon exposure to higher pressures, but viable CFUs were still present at 1500 MPa (1.5 GPa). The observation that even a small proportion of individuals can survive such extreme pressurization conditions is remarkable, that has only been suggested previously by the pioneering study of Sharma et al. (2002). These authors showed spectroscopic evidence for respiration by *in situ* diamond anvil cell studies of a similar strain exposed to pressures up to 1680 MPa. However, they also found that *E. coli* in its wild type state showed similar metabolic activity over the same pressure range, whereas our recent pressurization-cultivation experiments found no detectable CFUs among samples exposed to above 750 MPa (Vanlint et al., 2011). The rate of pressure drop in survivability between 250 and 500 MPa found for WT *E. coli* was much lower, however, decreasing by only 2.5 log (*N*) units over this range. It is obvious that the rate of decrease in bacterial survivability with exposure to extreme pressure stresses is highly species dependent. Wuytack et al. (2002) tested the survivability of 11 bacterial strains at pressures up to 400 MPa. They found that all microbes showed a similar large increase in cell death above 250 MPa, and they noted that Gram-negative bacteria (including *Shewanella*) appeared to show a lowered aptitude for high pressure survival than the Gram-positive examples included in their sample. These results raise interesting questions concerning the role of cellular structure in determining the degree of survival into extreme pressure ranges, among populations that have become adapted to live and grow under particular sets of environmental conditions.

Our second principal result concerns the high degree of survivability developed among randomly selected parts of surviving populations that had been resuscitated following initial exposure to high pressure treatments. In extensive studies of *E. coli* exposed to pressures up to 700 MPa, food technology researchers have identified genetic factors that may contribute to extreme pressure resistance (Hauben et al., 1997; Aertsen et al., 2004; Vanlint et al., 2013a,b). These authors reported an up regulation in RpoS activity compared with the parent strain, that could imply that genetic mutations play an important factor in developing pressure resistance (Griffin et al., 2011; Vanlint et al., 2013a,b; Meersman and McMillan, 2014). An independent study on *L. monocytogenes* shows an ability to mutate various protein sequences can allow the presence of a certain number of cells that are resistant to stress, even within the wild type population. This would then enable a subpopulation to survive under various stress conditions (Karatzas et al., 2005). However, in our present study of *S. oneidensis* it is known that only 1–2 doubling events should be expected during the 15 min experimental run time (Abboud et al., 2005), assuming that ambient pressure doubling rates apply. It is documented that bacterial metabolism slows considerably as a function of pressure implying that a reduction in growth rate would be expected (Picard et al., 2012). It thus seems unlikely that genetic modifications within individual cells would give rise to the observed pressure resistance acquired by selected elements of the population in our laboratory studies.

Studies have suggested that various biochemical effects can be implicated in extremophile survival strategies including changes in intracellular salt content and cell wall biochemistry (Bartlett, 2002; Griffin et al., 2011; Kish et al., 2012; Meersman et al., 2013), as well as other factors that have been identified as important in defining cellular integrity at high pressure (Allen et al., 1999; Allen and Bartlett, 2000; Schuster and Sleytr, 2002; Kawamoto et al., 2011; Picard and Daniel, 2014). It has been noted recently that lipid bilayer structures are templated on peptidoglycan layers such as those that form the outer cell wall of Gram-negative bacteria that could suggest a link to proteomics effects among the pressure-resistant parts of the population (Subramaniam et al., 2013).

We did not observe any clear cross-correlation between T- and P-response effects affecting survival in *S. oneidensis*. That result mirrors our previous report for *E. coli*, where no obvious correlation between high pressure and high temperature adaptability could be detected (Vanlint et al., 2011). Hauben et al. (1997) similarly concluded that there is no evidence for a correlation between pressure and temperature resistance, or indeed any common inactivation mechanism.

However, some studies have indicated evidence that such a P,T bacterial survival correlation does exist (Iwahashi et al., 1993; Benito et al., 1999; Balny et al., 2002), that is critically important for the food industry (Cheftel, 1995). Aertsen et al. (2004) showed 100× higher survival for bacteria that were exposed to heat shock prior to pressure exposure than those cells that were not heat shocked first. They suggested that an increase in the heat shock protein (HSP) *DnaK* expression is key to the bacterial cell survival under the combined stress conditions. For *S. oneidensis*, Yin and Gao (2011) proposed that the appearance of HSP and other stress-related proteins share some common features (Guo et al., 2004, 2006). In both cases ∼15% of the total genes experience a significant change over a 25 min period, that is similar to the 15 min timescales over which our P,T-exposure experiments were conducted. However, other studies have found no obvious correlations between HSPs and the emergence of pressure-resistance (Hartl, 1996; Feder and Hofmann, 1999; Hartl et al., 2011).

Further studies using libraries constituted from experimentally controlled pressure-adapted strains such are those produced here will be essential for developing our understanding of bacterial survival mechanisms in such extreme stress environments.

## CONCLUSION

Our data clearly show that fractions of an initial wild type *S. oneidensis* population survive following exposures to pressures extending into the gigapascal range. As found previously for *E. coli* (Vanlint et al., 2011), the survival improves if progressive increasing pressure stresses are applied to populations that have been resuscitated and cultivated from survivors of previous exposure to high pressure conditions. It is not clear if the “directed evolution” process involves a genetic, proteomic, or other biochemical response among the general population or simple selection of a small proportion of pre-conditioned survivors. However, the recovered samples have the ability to form colonies following recovery to ambient conditions. Our results indicate the survival for both WT and pressure adapted populations decreases rapidly following exposure to pressures in the 250–500 MPa range. The decrease in colony forming activity among the survivors appears to occur more rapidly for *S. oneidensis* than previously found for *E. coli*. There does not appear to be any obvious cross-correlation between the acquisition of pressure- and temperature resistance among the populations, except perhaps at the lowest temperatures where growth kinetics are minimized. *S. oneidensis* populations previously exposed to 1 GPa pressure exhibit substantially lower numbers of CFUs/ml following standard counting protocol conditions. These results fully demonstrate that bacterial survival following exposure to pressures into the GPa range can occur, but the survival mechanisms are not well established. Pursuing future studies of the bacterial genome, proteomics and cellular biochemistry and mechanisms both *in situ* and from pressure isolates from such laboratory studies will provide essential information for understanding the existence and function of organisms that survive under extreme high pressure and other applied environmental conditions.

## Conflict of Interest Statement

The authors declare that the research was conducted in the absence of any commercial or financial relationships that could be construed as a potential conflict of interest.
